# Maternal and infant health during the COVID-19 pandemic – A Pennsylvania Study Protocol

**DOI:** 10.1371/journal.pone.0323891

**Published:** 2025-05-15

**Authors:** Kristin K. Sznajder, Richard S. Legro, Douglas Teti, A. Dhanya Mackeen, Wadia Mulla, Hyagriv Simhan, Wenke Hwang

**Affiliations:** 1 Department of Public Health Sciences, The Pennsylvania State University College of Medicine, Hershey, Pennsylvania, United States of America; 2 Department of Obstetrics and Gynecology, The Pennsylvania State University College of Medicine, Hershey, Pennsylvania, United States of America; 3 Department of Human Development and Family Studies, The Pennsylvania State University, University Park, Pennsylvania, United States of America; 4 Division of Maternal-Fetal Medicine, Department of Obstetrics and Gynecology, Geisinger, Danville, Pennsylvania, United States of America; 5 Temple University Lewis Katz School of Medicine, Philadelphia, Pennsylvania, United States of America; 6 Magee-Womens Research Institute, University of Pittsburgh School of Medicine, Pittsburgh, Pennsylvania, United States of America; State University of Campinas: Universidade Estadual de Campinas, BRAZIL

## Abstract

**Background:**

Pregnant people are vulnerable to more severe outcomes of COVID-19 compared with their non-pregnant counterparts. Research is needed to systematically test the degree to which COVID-19 during pregnancy increases the risk for adverse maternal, perinatal, and infant health and development outcomes and whether social determinants of health or psychological/psychosocial health outcomes confound or intensify the risk. This protocol paper describes a prospective cohort study using electronic health record (EHR) and patient-reported data from four large health systems in Pennsylvania to examine neighborhood, social, and health factors predicting COVID-19 and its severity, birth weight, gestational age, and vaccination among pregnant people to 12 months postpartum.

**Methods:**

Our study will be conducted with two aims. Aim 1 will combine maternal and infant clinical data and neighborhood data from four health systems in Pennsylvania participating in a PCORI-supported clinical research network. The cohort will include all people who were pregnant between June 2019 and May 2025, along with linkage to their newborn delivery records. In Aim 2, a subset of pregnant people from the Aim 1 cohort will be recruited to participate in a series of surveys from pregnancy to one year postpartum. Survey instruments will be developed to collect patient-reported health and social information as well as patient-centered outcomes depicting whether and how the COVID-19 pandemic is impacting pregnant people and their newborns. Survey data will be collected during pregnancy and at one, six, and 12 months postpartum. Survey data will be linked with data from Aim 1 for analysis.

**Results:**

Ethical approval has been obtained at all sites. Subcontracts and data use agreements have been established. EHR data across health systems are being collected and curated. Surveys have been developed and recruitment and retention procedures implemented. Recruitment for the survey aim of the study began in July 2023 and is ongoing.

**Discussion:**

This study will advance multi-site research involving pregnant people across diverse communities in a time of public health crisis. Data from this study will provide additional evidence of the impact of the COVID-19 pandemic on pregnant people and their infants. Findings will help guide future clinical and public health practices in pandemics for pregnant people.

## Background

In the United States, pregnant people from racial and ethnic minority groups have endured worse health outcomes due to COVID-19 than white non-Hispanic people and have the highest incidence of adverse perinatal outcomes including hypertensive disorders, preterm birth (<37 weeks’ gestation), low birth weight, and maternal and neonatal mortality [1–3]. Previous research has demonstrated that pregnant individuals with COVID-19 have a higher risk of ICU admission, mechanical ventilation, and maternal mortality compared to those without COVID-19 [4–6]. Moreover, studies indicate that infants born to people with COVID-19 during pregnancy may be at greater risk for preterm birth, low birth weight, and NICU admission, which are not only adverse birth outcomes but also indicators for delayed child development which could signify the long term effects of COVID-19 during pregnancy [4–11]. However, much of this research has focused on clinical outcomes without integrating social determinants of health (SDOH) or psychological distress, leaving gaps in understanding the broader health impact of COVID-19 on perinatal health.

Pregnant people from racial and ethnic minority groups are more likely to be exposed to adverse social determinants of health (SDOH) that could increase their vulnerability to poor health outcomes such as poverty, food insecurity, unstable housing, rurality/urbanity, and limited access to healthcare, as well as psychological distress. These factors not only increase baseline health risks but have been shown to amplify the effects of COVID-19, exacerbating disparities in maternal and infant health [12–14]. During the pandemic, disruptions in prenatal care and differential access to COVID-19 treatment further worsened outcomes for marginalized populations [15–16]. For example, studies have reported lower vaccination rates among pregnant individuals from racial and ethnic minority groups, contributing to higher rates of severe illness [17]. Each of these SDOH factors may directly contribute to poor maternal and infant health outcomes [12]. Moreover, the intersection between SDOH and COVID-19 may further exacerbate poor health for both pregnant people and their infants [3,18–28].

COVID-19 and adverse pregnancy outcomes both cluster in minority populations; however, there is a dearth of information on whether SDOH or psychological distress confounds or moderates the association between COVID-19 during pregnancy and maternal and infant health outcomes [29–36]. Few studies have examined how individual- and neighborhood- level social determinants of health influence COVID-19-related maternal and infant outcomes [15,37,38]. Moreover, most research has relied on either patient-reported data or electronic health records (EHR), rather than integrating these sources to provide a comprehensive understanding of COVID-19’s impact on perinatal health. While evidence from studies on the association between COVID-19 and maternal, perinatal, and infant health outcomes continue to emerge, results are mixed or inconclusive [6]. This study fills these critical gaps by integrating EHRs with patient-reported data to examine how COVID-19 and the broader pandemic affects the lives of pregnant people and their infants clinically, socially, and mentally. The study design will also allow researchers to assess specific mechanisms as to how SDOH can contribute health disparities.

Research with larger samples integrating self-report and EHR data is urgently needed to understand the effects the COVID-19 pandemic has had on maternal and infant health. This project will systematically test the degree to which COVID-19 and the COVID-19 pandemic increases the risk for adverse maternal, perinatal, and infant development outcomes and whether SDOH or psychological/psychosocial health outcomes confound or intensify the association. Furthermore, this study will estimate the contribution of SDOH and psychological distress as moderators in the causal pathway of the association between COVID-19 and maternal and infant health outcomes, as well as provide further evidence on the prevalence of adverse health outcomes associated with COVID-19 for pregnant and postpartum people and their infants.

### Objectives

This protocol paper describes a prospective cohort study examining neighborhood, social, and health factors predicting COVID-19, COVID-19 severity, birth weight, gestational age, and vaccination among people during pregnancy until 12 months postpartum and their infants.

The study has four key objectives outlined in the protocol.

1. Identify individual- and neighborhood-level risk factors for COVID-19 and COVID-19 severity in pregnant people and related maternal and infant health outcomes.
2. Estimate the association between COVID-19 and COVID-19 severity on maternal, perinatal, and infant health outcomes.
3. Assess factors associated with pregnant people receiving the COVID-19 vaccine and the maternal, perinatal, and infant health outcomes associated with receiving the COVID-19 vaccine.
4. Investigate the association between psychological distress, resilience, COVID-19 during pregnancy, and adverse maternal, perinatal, and infant health and development outcomes.

## Materials and methods

### Study design

This study has two aims that will be conducted simultaneously.

### Aim 1

A cohort study on all pregnant people from June 2019 to May 2025 at each of the four study sites in Pennsylvania will be completed to examine factors associated with COVID-19, COVID-19 severity, birth weight, gestational age, and vaccination. All four sites are participating institutions in a PCORI-supported Clinical Research Network which standardizes and harmonizes EHRs from disparate sources to conform to the PCORnet Common Data Model (CDM) [39,40]. EHRs for all pregnant people at each health system will be combined and linked with their infant’s EHR. Specifically, variables in the research databases will include age, race, ethnicity, prescription drug histories, COVID-19 diagnoses, comorbidities, hospitalizations, pregnancy complications, and geographic-level linkage keys based on census block group and/or zip code in order to integrate an Area Deprivation Index (ADI). The ADI is a standardized ranking of geographic-based neighborhood at the census block group level by their relative socioeconomic disadvantage compared with the nation and state and ranges from one to 100, with one indicating the lowest level of deprivation and 100 indicating the highest level [41]. Data from all pregnant people will be included for two years before their pregnancy to ensure important comorbidities are included and up to 12 months postpartum for the pregnant person and their infant. While the PCORnet CDM includes data on diagnoses, encounters, and prescriptions, the PCORI Common Data Model does not reliably include maternal and neonatal data including intensive care unit (ICU) or neonatal ICU (NICU) admission, birth weight, gestational age, breastfeeding, preterm birth, head circumference, as well as mother-child linkages. A maternal and child health (MCH) data module will be developed by each health system to incorporate these critical maternal and neonatal health elements. This module will need to be harmonized across sites. We will review the data at each site for format, structure, and units. We expect data to be sent to us in multiple formats and we will create one data table for each site inclusive of all variables and matching mother and newborn with links to the CDM. Any errors, duplicates, or inconsistencies will be identified and we will ensure data validity and reliability through reviewing the distribution of the variables and checking the data against external sources. The harmonization process will be documented for each site.

### Aim 2

A survey will be conducted through REDCap [42] among a subset of pregnant people recruited using Aim 1 data as our sampling frame. An open cohort design will be implemented to prospectively recruit people during their pregnancy. People will be recruited during any point in their pregnancy and will complete a baseline survey upon recruitment, undergo monthly check-ins to inquire if their child was born and their child’s birthdate, complete a survey at one-month postpartum for detailed information on the birth experience, and complete surveys at six- and 12- months postpartum to measure infant health and development and maternal health over time. Each site will ascertain a list of currently pregnant people from its own EHR by extracting data at least once every three months to align with routine data updates and investigators will create a recruitment list from these data which will include all pregnant people, selected by the presence of a pregnancy ICD-10 code and absence of an ICD-10 code indicating the termination or completion of pregnancy. The recruitment list will oversample people of racial and ethnic minority groups, people living in rural areas, defined by RUCA code, and people with COVID-19. The recruitment list will be returned to each site for recruitment. Before contacting potential participants, the pregnancy will be validated in each site’s EHR system to ensure people who are no longer pregnant are not contacted. Using a site-specific REDCap module, each site will have access to its own patient list and will contact people for recruitment through emails, letters, phone calls, portal messages, in-person, and text messages. Participants will receive compensation, in the form of a gift card, for each of the four surveys they complete at increasing amounts (monthly check-ins during pregnancy will not provide compensation). Aim 1 data including EHR data, data from the MCH module, and ADI data will be linked with survey data. Table 1 outlines the data sources that will be used in our analyses. Our survey questions include questions on sleep, nutrition, physical activity, COVID-19 symptoms, vaccination, maternal mental well-being health, parenting, infant development, maternal responsiveness, and stress (S1 Table). Most scales are commonly used, validated scales and the questions developed for this study were created with the appropriate face validity.

**Table 1:** Inclusion criteria and data sources.

Aim	Inclusion Criteria	Exclusion Criteria	Data Sources
Aim 1	11 years to 55 years of age Received care at one of four Pennsylvania academic health systems included in this study At least one pregnancy since June 2019	Under 11 or over 55 years of age Not receiving care at one of the four Pennsylvania academic health systems included in this study No pregnancies since June 2019	PCORI Common Data Model MCH Module ADI
Aim 2	18 years or over Able to understand English or Spanish Pregnant at the time of enrollment Able to consent Included in Aim 1 Not planning to place their baby for adoption Not serving as a surrogate	Under 18 Not able to understand English or Spanish Not pregnant at the time of enrollment Unable to consent Planning to place their baby for adoption Serving as a surrogate Not included in Aim 1	Aim 1 data Baseline survey 1-month survey 6-month survey 12-month survey

### Study sites

This study will leverage an existing PCORnet research network with a history of successful large-scale research projects, national visibility, and an existing CDM [39,43,44]. The study sites are four academic medical systems in Pennsylvania and together cover all counties in the state. The PCORI network has conformed most of the EHR data to a national standard PCORnet common data model in which, all participating sites extract, format, and structure data uniformly to ensure consistency and readiness for multi-site studies. The data are curated quarterly and monitored by the PCORnet Coordinating Center, for data quality assurance.

### Data flow

**Fig 1:**
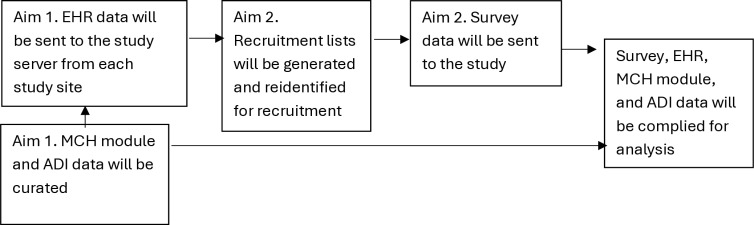
Data flow for Aim 1 and Aim 2.

EHR data from each study site will be sent at least quarterly to our study server and will make up the sampling frame for Aim 2 recruitment. Recruitment lists will be generated using these EHR data and then reidentified at each site for recruitment. Data in the MCH module and ADI data will be gathered at least once at the beginning of the study and once at the end of the study. A diagram of our data flow is illustrated in Fig 1.

### Study population

#### Aim 1.

People between 11 years of age and 55 years of age, who receive care at one of four academic health systems in Pennsylvania, the University of Pittsburgh Medical Center, Geisinger Health, Temple Health, and Penn State Health, and had at least one pregnancy since June 2019 are eligible for inclusion into the EHR cohort (Table 1).

#### Aim 2.

From those in the Aim 1 cohort, people who are at least 18 years of age, pregnant at the time of recruitment, not planning to place their child for adoption, not serving as a surrogate, and able to understand English or Spanish are eligible for the survey component of the study (Table 1). The sub-cohort that will be selected to participate in the survey will be a stratified random sample of pregnant people from data compiled from the four EHRs eligible in the larger EHR population.

### Ethnical approval

This study has been approved by the institutional review boards of each collaborating institution including The Pennsylvania State University, Temple Health, Geisinger Health, and the University of Pittsburgh and the funder of this project, the Pennsylvania Department of Health. In Aim 1, authors will not have access to information that could identify individual participants during or after data collection. In Aim 2, authors will have access to identifiable information for recruitment purposes which will only be available at the recruiting site. Identifiable data will not be shared to other sites or linked with data for analysis. A summary explanation of research will be provided to all individuals recruited in Aim 2 and electronic consent will be obtained by clicking the ‘submit’ button after reviewing the consent document in REDCap before participants will be able to enroll in the study.

### Power analysis

#### Aim 1.

A sample size of 24,000 would allow us to assess the association between COVID-19 and hypertensive disorders during pregnancy with an odds ratio of 1.27 and 23% hypertensive disorders and a 5% diagnosis rate of COVID-19, with 80% power and alpha of 0.05 and a variance inflation factor or two [45–46].

#### Aim 2.

Using data estimates on the association between COVID-19 during pregnancy and preterm birth, an estimated 9.8% of people in Pennsylvania will have a preterm birth and COVID-19 will increase the odds for preterm birth by 1.82 [47]. With 80% power, an alpha of 0.05, and a variance inflation factor of two, a sample size of 500 is required [45,46]. After considering a 20% dropout rate, we aim to enroll 600 pregnant people for analysis.

### Statistical analysis

#### Aim 1.

We will conduct multi-level spatial and fixed effects regression modeling to assess our key dependent variables including COVID-19, COVID-19 severity, hypertensive disorders, birth weight, gestational age, and vaccination. SDOH data at the neighborhood- and individual-level, and clinical comorbidities will be independent variables. We will adjust for possible confounders in the regression models and we intend to further assess the mediation or moderation effect of SDOH [48]. For potential confounding variables, we will first perform a series of univariate analyses for screening and then incorporate significant variables, as well as hypothesized confounders into multiple regression models. As a means to check the model fit, comparing the AIC/BIC between stepwise regression and regression models using theory-drive variable selection will be further applied to optimize our models.

#### Aim 2.

We will conduct multi-level spatial and fixed effects regression modeling to assess the association between COVID-19 and psychological distress controlling for SDOH and clinical variables. Additionally, an interaction term for COVID-19 and psychological distress will be included in multi-level spatial models to test for effect modification and to control for individual- and neighborhood-level SDOH for the outcomes of birth weight and gestational age, or whether the psychological distress intensifies the association between COVID-19 and birth weight and gestational age [48]. Further, multiple regression models will be used to assess whether resilience is associated with social support and whether resilience and/or social support is associated with lower levels of maternal psychological distress. Whether resilience or social support mitigates the association between COVID-19 (independent variable) and maternal psychological distress (dependent variable) will be evaluated [48].

#### Loss to follow up and missingness.

In Aim 2, survey data may be missing or incomplete or participants may be lost to follow up. To address missing data for key covariates such as SDOH, demographic factors, and comorbidities, we will employ multiple imputation techniques using SAS procedures PROC MI and PROC MIANALYZE. Diagnostic checks for imputation will include convergence evaluations, distributional comparisons, and predictive mean matching to ensure the validity and reliability of the imputed datasets. Every attempt will be made to contact survey participants and obtain the missing information, and reasons for withdrawal or non-participation will be documented. For self-reported outcomes with more than 20% item non-responses, inverse probability weighting may be applied to adjust for potential biases arising from differential participation and to improve the validity of the results.

#### Subgroup analyses.

We will perform subgroup analyses, which will provide critical insights into maternal health disparities in both our Aim 1 and Aim 2 study populations. The subgroup analyses will assess differences in outcomes and exposures by race, ethnicity, socioeconomic status, and geographic region.

#### Statement on data sharing.

Our data are saved at on institutional repository. At present, the infrastructure to share deidentified data has not be established and we are bound by current data use agreements. We will consider avenues for data sharing to external investigators in the future commiserate with our publications policy.

## Results

### Aim 1

EHR data were initially accessible beginning on July 18, 2023. Data collection and curation is ongoing and is projected to continue through the end of May 2025.

### Aim 2

Recruitment for the survey component of this study began on July 18, 2023 and is ongoing.

## Discussion

Data are inconclusive on what factors are associated with COVID-19 during pregnancy and why some pregnant people have more severe COVID-19 than others. Few studies have combined neighborhood-level data with individual-level data from EHRs to illustrate the SDOH associated with COVID-19 and COVID-19 severity [49–50]. Research from this study will contribute to the continued development of clinical guidelines and public health disease prevention and resource allocation efforts for addressing the COVID-19 pandemic and future pandemics.

### Strengths

The key strengths of this study are its longitudinal design and its combination of EHR and ADI data and its representativeness across the state of Pennsylvania. An additional strength is the study’s integration of the EHR and ADI data with survey data. The triangulation of multiple data sources will allow for a more nuanced understanding of how COVID-19 and the COVID-19 pandemic has affected pregnant people and their infants.

### Limitations

In Aim 2, we rely on self-report for variables including psychological distress and social factors which could introduce recall and social desirability bias. To mitigate this, we will use validated survey instruments and will compare self-reported data with available objective measures from the EHR when feasible. Additionally, it is possible that the study is vulnerable to selection bias in Aim 2 including those volunteering to participate and those retained throughout the year postpartum. To address this, we will carefully track recruitment and retention patterns, compare baseline characteristics of those recruited, enrolled, and lost to follow-up, and conduct sensitivity analyses to assess the impact of enrollment and attrition. While we aim comprehensively account for confounders, residual confounding remains a possibility and could impact our findings. To address this, we will use directed acyclic graphs to clarify potential sources of bias and apply statistical techniques (e.g., propensity scores, E-values) to evaluate the robustness of our conclusions. Finally, there could be temporal influences in our cohort including shifts in public health policies, access to healthcare, and vaccination guidelines. We will account for known temporal changes in our analyses and conduct subgroup analyses to explore the potential impact of major policy shifts. However, we acknowledge that unknown changes may still occur, which we will address in our interpretation of results.

### Conclusions

At the completion of this project, we will have dramatically improved our understanding of the impact of COVID-19 during pregnancy on maternal and infant health, risk factors associated with disparities in COVID-19 prognosis, and psychological distress associated with the COVID-19 pandemic and associated maternal and infant health outcomes. The evidence gained from this study is expected to help guide clinical practice, public health practice, community outreach, and inform future research and public health policy.

## Supporting information

S1 Table Patient-centered survey questions for all survey time points. (DOCX)
