# Supplementary material for: Maternal and infant health during the COVID-19 pandemic – A Pennsylvania Study Protocol
Source: PLoS One. 2025 May 15;20(5):e0323891. doi: 10.1371/journal.pone.0323891 (PMC12080819; doi:10.1371/journal.pone.0323891)
Supplement: S1 Table — (DOCX) [file pone.0323891.s001.docx]

| **Supplemental Table 1. Patient-centered Survey Questions for All Survey Time Points** | |
| --- | --- |
| **Perinatal/Infant Outcomes** | **Measure** |
| SARS-CoV-2 positive test ^B, 1, 6, 12^ | Self-report including date of diagnosis |
| COVID-19 vaccination ^B, 1, 6, 12^ | Self-report including type of vaccine and date of vaccine |
| Maternal sleep ^B, 1, 6, 12^ | Pittsburgh Sleep Quality Index [51] |
| Nutrition ^B, 1, 6, 12^ | Michigan Behavioral Risk Factor Surveillance System Nutrition Scale [52] |
| Physical Activity ^B, 1, 6, 12^ | NHANES Physical Activity Questionnaire [53] |
| Health Behaviors ^B, 1, 6, 12^ | Questions on contact patterns, job-related exposures, and uptake of preventive behaviors (e.g., masks and social distancing) from preliminary study based on the COVID-19 and Perinatal Experiences Study [54] and the PRAMS COVID-19 Experiences Questions [55] |
| Access to care ^B, 1, 6, 12^ | Questions on geographic or financial difficulty associated with accessing care, fear to access care due to the COVID-19 pandemic, or lack of available healthcare or COVID-19 from preliminary study |
| Symptoms of COVID-19 ^B, 1, 6, 12^ | Symptom scale developed in preliminary study |
| Depressive Symptoms ^B, 1, 6, 12^ | Edinburgh Postnatal Depression Scale [56-58] |
| Anxiety ^B, 1, 6, 12^ | General Anxiety Disorder-7 (GAD-7) [59] |
| Substance abuse ^B, 1, 6, 12^ | NIDA-Modified Alcohol, Smoking, and Substance Involvement Screening Test (NM ASSIST) [60] |
| Vaccine Hesitancy ^B, 1, 6, 12^ | Vaccine Hesitancy Scale [61] |
| Domestic violence ^B, 1, 6, 12^ | Specific survey question |
| Financial Strain ^B, 1, 6, 12^ | A scale on financial stressors such as unemployment developed in preliminary study |
| Impact of COVID-19 on daily life among household and extended family/friends. ^B, 1, 6, 12^ | Coronavirus Impact Scale [62] |
| Social Support ^B, 1, 6, 12^ | Medical Outcomes Study Social Support Survey [63] |
| Resilience ^B, 1, 6, 12^ | Brief Resilience Scale (BRS) [64] |
| Mental Well-being during Pregnancy ^B^ | Tilburg Pregnancy Distress Scale [65]  Pregnancy-related Anxiety Scale [66]  Pandemic-related Pregnancy Stress Scale [67] |
| Stress ^B, 1, 6, 12^ | Perceived Stress Scale [68]  Psychosocial Hassles Scale [69] |
| Parenting ^1, 6, 12^ | Parenting Sense of Competence Scale[70]  Co-parenting Relationship Scale [71] |
| Maternal Responsiveness ^1, 6, 12^ | Maternal Responsiveness Questionnaire (MRQ) [72] |
| Feelings towards infant crying ^1, 6, 12^ | My Emotions Questionnaire [73] |
| Child development ^6, 12^ | Ages & Stages Questionnaire, Third Edition (ASQ-3) [74]  Ages & Stages Questionnaires: Social-Emotional, Second Edition (ASQ-SE-2) [75] |
| Infant self-regulation/temperament ^1, 6, 12^ | Infant Behavior Questionnaire–Revised (IBQ-R) [76] |
| Infant sleep ^1, 6, 12^ | Sleep Practices Questionnaire (SPQ) [77-78] |

^B^ Collected at Baseline, ^1^ Collected at one month, ^6^ Collected at six months, ^12^ Collected at 12 months

51. Buysse DJ, Reynolds CF, 3rd, Monk TH, Berman SR, Kupfer DJ. The Pittsburgh Sleep Quality Index: a new instrument for psychiatric practice and research. *Psychiatry research*. May 1989;28(2):193-213. doi:10.1016/0165-1781(89)90047-4

52. Rafferty AP, Anderson JV, McGee HB, Miller CE. A healthy diet indicator: quantifying compliance with the dietary guidelines using the BRFSS. *Preventive medicine*. 2002;35(1):9-15.

53. Evenson KR, Wen F. National trends in self-reported physical activity and sedentary behaviors among pregnant women: NHANES 1999–2006. *Preventive medicine*. 2010;50(3):123-128.

54. Thomason ME, Graham A, VanTieghem MR. COPE: Coronavirus Perinatal Experiences -Impact Survey(COPE-IS). Accessed July 21, 2021. <https://osf.io/h9q5p/>

55. Centers for Disease Control and Prevention. PRAMS: COVID-19 Experiences Questions. Accessed July 21, 2021. <https://www.cdc.gov/prams/questionnaire.htm>

56. Murray D, Cox JL. Screening for depression during pregnancy with the Edinburgh Depression Scale (EDDS). *Journal of Reproductive and Infant Psychology*. 1990;8(2):99-107.

57. Cox J, Holden J. *Perinatal mental health: A guide to the Edinburgh Postnatal Depression Scale (EPDS)*. Royal College of Psychiatrists; 2003.

58. Cox JL, Chapman G, Murray D, Jones P. Validation of the Edinburgh Postnatal Depression Scale (EPDS) in non-postnatal women. *Journal of affective disorders*. 1996;39(3):185-189.

59. Spitzer RL, Kroenke K, Williams JB, Löwe B. A brief measure for assessing generalized anxiety disorder: the GAD-7. *Archives of Internal Medicine*. 2006;166(10):1092-1097.

60. Oga EA, Mark K, Peters EN, Coleman-Cowger VH. Validation of the NIDA-modified ASSIST as a screening tool for prenatal drug use in an urban setting in the United States. *Journal of Addiction Medicine*. 2020;

61. Akel KB, Masters NB, Shih S-F, Lu Y, Wagner AL. Modification of a vaccine hesitancy scale for use in adult vaccinations in the United States and China. *Human Vaccines & Immunotherapeutics*. 2021:1-8.

62. Kaufman J, Stoddard J. The Coronavirus Impact Scale. *Retreived from* [*https://disasterinfo*](https://disasterinfo) *nlm nih gov/search*. 2020;

63. Moser A, Stuck AE, Silliman RA, Ganz PA, Clough-Gorr KM. The eight-item modified Medical Outcomes Study Social Support Survey: psychometric evaluation showed excellent performance. *Journal of Clinical Epidemiology*. 2012;65(10):1107-1116.

64. Smith BW, Dalen J, Wiggins K, Tooley E, Christopher P, Bernard J. The brief resilience scale: assessing the ability to bounce back. *International Journal of Behavioral Medicine*. 2008;15(3):194-200.

65. Pop VJ, Pommer AM, Pop-Purceleanu M, Wijnen HA, Bergink V, Pouwer F. Development of the Tilburg pregnancy distress scale: the TPDS. *BMC pregnancy and childbirth*. 2011;11(1):1-8.

66. Brunton RJ, Dryer R, Saliba A, Kohlhoff J. The initial development of the Pregnancy-related Anxiety Scale. *Women and Birth*. 2019/02/01/ 2019;32(1):e118-e130. doi:<https://doi.org/10.1016/j.wombi.2018.05.004>

67. Preis H, Mahaffey B, Lobel M. Psychometric properties of the pandemic-related pregnancy stress scale (PREPS). *Journal of Psychosomatic Obstetrics & Gynecology*. 2020;41(3):191-197.

68. Nielsen MG, Ørnbøl E, Vestergaard M, et al. The construct validity of the Perceived Stress Scale. *Journal of Psychosomatic Research*. 2016;84:22-30.

69. Misra DP, O'Campo P, Strobino D. Testing a sociomedical model for preterm delivery. *Paediatric and perinatal epidemiology*. 2001;15(2):110-122.

70. Ohan JL, Leung DW, Johnston C. The Parenting Sense of Competence scale: Evidence of a stable factor structure and validity. *Canadian Journal of Behavioural Science/Revue Canadienne des Sciences du Comportement*. 2000;32(4):251.

71. Feinberg ME, Brown LD, Kan ML. A multi-domain self-report measure of coparenting. *Parenting*. 2012;12(1):1-21.

72. Leerkes E, Qu J. The maternal (non) responsiveness questionnaire: Initial factor structure and validation. *Infant and Child Development*. 2017;26(3):e1992.

73. Leerkes EM, Qu J. The My Emotions Questionnaire: A self‐report of mothers’ emotional responses to infant crying. *Infant Mental Health Journal*. 2020;41(1):94-107.

74. Squires J, Twombly E, Bricker D, Potter L. *The ASQ-3 user's guide (3rd ed.)*. 2009.

75. Squires J, Bricker D, Twombly E. Ages and stages questionnaires: Social-emotional. *Baltimore: Brookes*. 2002;

76. Parade SH, Leerkes EM. The reliability and validity of the Infant Behavior Questionnaire-Revised. *Infant Behavior and Development*. 2008;31(4):637-646.

77. Teti DM, Kim B-R, Mayer G, Countermine M. Maternal emotional availability at bedtime predicts infant sleep quality. *Journal of Family Psychology*. 2010;24(3):307.

78. Goldberg WA, Keller MA. Parent–infant co‐sleeping: why the interest and concern? *Infant and Child Development: An International Journal of Research and Practice*. 2007;16(4):331-339.
